# Small molecules targeting glycogen synthase kinase 3 as potential drug candidates for the treatment of retinitis pigmentosa

**DOI:** 10.1080/14756366.2016.1265522

**Published:** 2017-01-23

**Authors:** Miguel Marchena, Beatriz Villarejo-Zori, Josefa Zaldivar-Diez, Valle Palomo, Carmen Gil, Catalina Hernández-Sánchez, Ana Martínez, Enrique J. de la Rosa

**Affiliations:** a Department of Cellular and Molecular Medicine, Centro de Investigaciones Biológicas (CSIC), Madrid, Spain;; b Department of Chemical and Physical Biology, Centro de Investigaciones Biológicas (CSIC), Madrid, Spain

**Keywords:** GSK-3 inhibitors, retinal diseases, retinitis pigmentosa, glaucoma

## Abstract

Retinitis pigmentosa (RP) is an inherited retinal dystrophy that courses with progressive degeneration of retinal tissue and loss of vision. Currently, RP is an unpreventable, incurable condition. We propose glycogen synthase kinase 3 (GSK-3) inhibitors as potential leads for retinal cell neuroprotection, since the retina is also a part of the central nervous system and GSK-3 inhibitors are potent neuroprotectant agents. Using a chemical genetic approach, diverse small molecules with different potency and binding mode to GSK-3 have been used to validate and confirm GSK-3 as a pharmacological target for RP. Moreover, this medicinal chemistry approach has provided new leads for the future disease-modifying treatment of RP.

## Introduction

Retinal neurodegenerative diseases like age-related macular degeneration, glaucoma, diabetic retinopathy and retinitis pigmentosa (RP) are disorders that have different etiology and pathogenesis and where the progressive impairment of diverse retinal cell types leads to vision loss. However, at the cellular and molecular level, the response to retinal injury is similar in all of them, and results in morphological and functional impairment of retinal cells. Interestingly, an inflammatory response, oxidative stress and activation of apoptotic pathways are common features in all these diseases^1^. Altogether they represent a major cause of blindness and a public health challenge. Up to date, there is not effective treatment for their neurodegenerative component, although many lines of basic research are devoted to identify their basic pathology and to develop effective treatments^2^.

Among other retinal degenerative disorders, RP and glaucoma course with neuronal death of photoreceptors and retinal ganglion cells, respectively. RP, together with other inherited retinal dystrophies, comprises a genetically heterogeneous, although clinically similar, group of neurodegenerative retinal disorders caused by mutations in more than 250 different genes. These genetic defects disrupt the function of photoreceptor cells which gradually causes night blindness, progressive constriction of the visual field and eventually blindness^3^. The progression of visual loss rate varies within individuals. Most people with RP are legally blind by the age of 40. RP is categorized as a rare disease, with a prevalence reported of one case out of the 3000–4000 individuals^4^.

The retina is a highly specialized sensory tissue that belongs to the central nervous system. It may be considered as an accessible, external part of the brain, as both derive from the most rostral neural tube^5^. In fact, the retina and the rest of the central nervous system share identical physiological and pathological molecular pathways. Consequently, diseases of the brain and the retina show similarities. For example, neuroinflammation appears as a relevant process for retina and brain neurodegenerative diseases^1^ ^,^ ^6^ and may provide a promising target when looking for therapeutic treatments for the central nervous system in general, and for the retina in particular^7^.

Glycogen synthase kinase 3 (GSK-3) is a constitutively active Ser/Thr kinase ubiquitously expressed in the human body. Although it was first identified as an enzyme involved in glycogen synthesis^8^, its involvement in the regulation of multiple physiological functions has been well-characterized. GSK-3 has been categorized as a central kinase in a large number of prevalent diseases such as psychiatric and neurodegenerative diseases, diabetes and cancer^9^. Inflammation has been postulated as a link for all these diseases-mediated by GSK-3 malfunction^10^. The recent discovery of cellular location of GSK-3 in retina, both in Müller and photoreceptor cells^11^, led to recently propose GSK-3 as a key player in retinal neuronal death both in early diabetic retinopathy and retinal ischemic injury^12^ ^,^ ^13^. Moreover, lithium which among many different targets is a weak GSK-3 inhibitor (IC_50 _=_ _2 mM) has been used to reduce intraocular pressure in a rat model of glaucoma when it was intraperitoneally administered^14^. Additionally, valproic acid, that has been reported as an indirect inhibitor of GSK-3 through direct inhibition of histone deacetylase^15^, showed a short-term benefit for RP patients^16^ ^,^ ^17^, although the mechanism underlying this effect is not well-understood^18^.

Based in our previous experience with GSK-3 inhibitors as drug candidates for neurodegenerative disorders^19^, and in the search for RP therapies, our goal is to study whether GSK-3 inhibitors could be systemically administered as a potential therapy for RP. In this communication, we report *ex vivo* studies that show the potential of GSK-3 inhibitors to protect retinal cells and, thus, their potential to be translated into treatments to delay vision loss, particularly in RP.

## Methods

### Chemistry

Compounds **1**, **2** and tideglusib have been synthesized in our laboratories following their previous described procedures^20^.

### Biology

#### Animals

The *rd10* mouse model of RP is a homozygous recessive mutant for phosphodiesterase 6b (*Pde6b^rd10/rd10^*) on a C57BL/6J background. It was kindly provided by Bo Chang from The Jackson Laboratory (Bar Harbor, ME). Wild type (WT) control mice of the same background were obtained from The Jackson Laboratory. All animals were housed and handled in accordance with the ARVO statement for the use of animals in ophthalmic and vision research, European Union guidelines, and those of the local ethics committees of the CSIC and the Comunidad de Madrid. Mice were bred in the CIB core facilities on a 12/12-h light/dark cycle.

#### Retina explant cultures

P23 *rd10* and P42–43 WT mice were euthanized, and their eyes were enucleated. Retinas were dissected and cultured on Teflon filters in R16 medium as described^21^ (See also Figure 2). For the glaucoma model, WT retinas were treated with 50 µM NMDA during 48 h, with a medium change after 24 h. The *rd10* mouse retinas were cultured for 24 h. Compound **1** was employed at 3.2 µM, compound **2** at 10 µM and tideglusib at 10 µM. Retinas were subsequently fixed in 4% (wt/vol) paraformaldehyde in phosphate buffer 0.1 M, pH 7.4 for 1 h at RT and processed for the detection of cell death.

#### Cell death visualization and counting

Ganglion cell and photoreceptor cell death was visualized by DNA fragmentation assay terminal deoxynucleotidyl transferase-mediated dUTP nick end labeling (DeadEnd Fluorometric TUNEL system; Promega, Madison, WI) as described^22^. After labeling, the retinas were mounted in Fluoromount-G (Southern Biotechnology, Birmingham, AL), stained with DAPI, and analyzed on a laser confocal microscope (TCS SP5; Leica, Microsystems, Wetzlar, Germany). Image acquisition was performed in four areas of each retina. Serial optical sections were acquired in the depth of the ganglion cell layer or the outer nuclear layer, as determined in *z*-axis retinal sections, to ensure that TUNEL-positive nuclei belong to ganglion cells or to photoreceptors, respectively. Direct counting of TUNEL-positive cells was carried out on merged images using ImageJ v1.48.s software. Paired statistical analysis was performed using the Wilcoxon signed-rank test.

## Results and discussion

**Figure 1.**
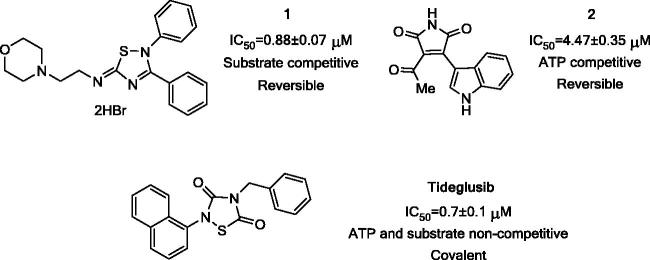
Chemical structures of the selected candidates and their GSK-3 inhibition features.

In order to confirm the therapeutic relevance of GSK-3 modulation on retinal pathologies, we employed a chemical genetic approach in *ex vivo* studies. The chemical genetic rationale postulates that different small chemical probes assayed in different *in vitro*, *ex vivo* and/or *in vivo* studies contribute to decipher the role of a potential therapeutic target^23^. Consequently, here we selected three chemically diverse small heterocyclic molecules designed and synthetized in our laboratory that target GSK-3 by different mechanism of inhibition (Figure 1): a substrate competitive inhibitor with an iminothiadiazole scaffold^1^ ^,^ ^24^ an ATP competitive inhibitor belonging to the maleimide heterocyclic family^2^ ^,^ ^25^ and tideglusib, a non-ATP, non-substrate competitive GSK-3 inhibitor currently in clinical trials for autism spectrum disorders^26^. Tideglusib is a thiadiazolidindione (TDZD) and currently the most advanced compound in clinical development among the selected GSK-3 inhibitors. Additionally, **1**, **2** and Tideglusib have previously been tested in cell cultures and animal models showing no toxicity^27–30^.

**Figure 2.**
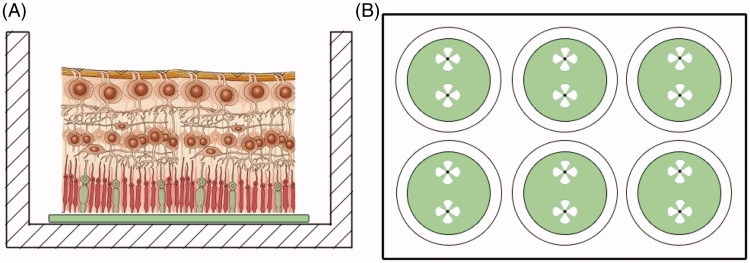
Organotypic culture design. (A) The retinas were mounted with the photoreceptors in direct contact to the Teflon disc. (B) After extraction from the eyeball, four cuts were made in the retina to facilitate attachment. Two retinas were cultured in each well.

First we assayed the two more novel inhibitors (**1** and **2**) in retinal explants obtained from *rd10* and cultured over Teflon discs (Millipore), as exemplified in Figure 2.

**Figure 3.**
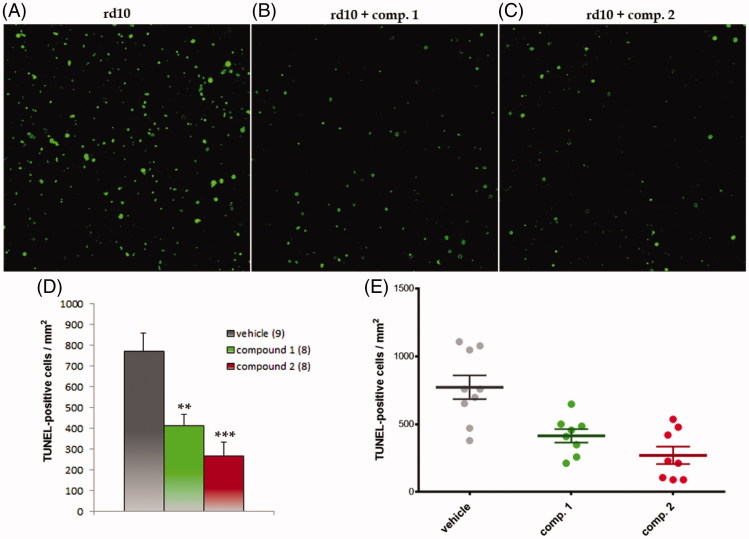
GSK-3 inhibitors decreased photoreceptor cell death in *rd10* mouse retinal explants. Representative images of groups (A) vehicle, (B) treatment with compound **1** and (C) treatment with compound **2**. D–E. Graphic representation of data: (D) mean ± standard error is represented for each experimental group. The number in brackets corresponds to the number of retinas; (E) Individual retinal values are depicted. Significances were calculated with *t* student test **: *p* < 0.01, ***: *p* < 0.001.

The *rd10* mouse retinal explants are a RP disease model in which there is intrinsic photoreceptor cell death. The retinas were dissected at postnatal day P23, at the peak of cell death^31^, and cultured in the absence or presence of compounds **1** and **2**. Cell death was visualized by TUNEL and quantified. Both GSK-3 inhibitors significantly reduced photoreceptor cell death (Figure 3) elicited a neuroprotective action in the RP model. Further, they suggest a novel potential role of GSK-3 inhibition on the treatment of this retinal pathology.

Next, the retinal explants were obtained from wild type (WT) mice and cultured as above in the presence of *N*-methyl-*D*-aspartate (NMDA)^32^, a glutamate receptor agonist. This excitotoxic treatment mimics glaucoma-related damage of the retinal ganglion cells and has been used to validate the potential of GSK-3 inhibitors in retinal cells protection.

**Figure 4.**
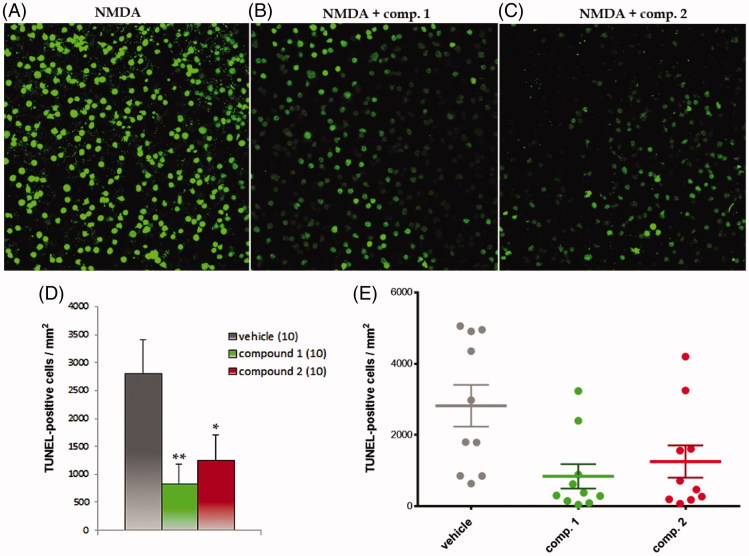
GSK-3 inhibitors decreased NMDA-induced retinal ganglion cell death. Representative images of groups (A) NMDA, (B) NMDA + compound **1** and (C) NMDA + compound **2**. D–E. Graphic representation of data: (D) mean ± standard error is represented for each experimental group. The number in brackets corresponds to the number of retinas; (E) Individual retinal values are depicted. Significances were calculated with *t* student test *: *p* < 0.05, **: *p* < 0.01.

NMDA treatment induced massive cell death in the ganglion cell layer that was significantly reduced when the retinas were co-incubated with either one of the two GSK-3 inhibitors (Figure 4). These results confirmed the neuroprotective effect of GSK-3 inhibition in retinal tissue, since the two pharmacological probes, with different chemical scaffolds but with the common property of being selective GSK-3 inhibitors, reduced significantly retinal cell death^33^.

**Figure 5.**
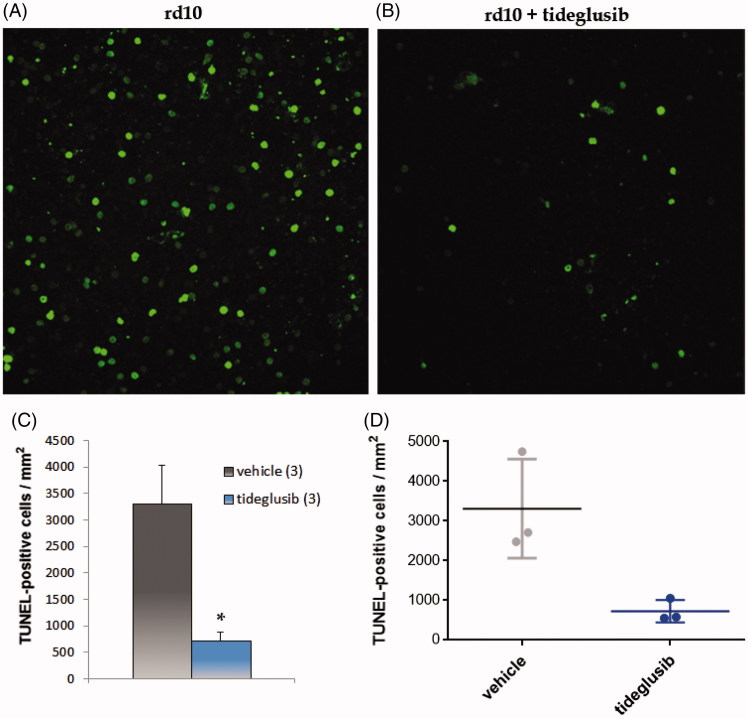
Tideglusib decreased photoreceptor cell death in *rd10* mouse retinal explants. Representative images of groups (A) vehicle, (B) tideglusib. C–D. Graphic representation of data: (C) mean ± standard error is represented for each experimental group. The number in brackets corresponds to the number of retinas; (D) Individual retinal values are depicted. Significances were calculated with *t* student test *: *p* < 0.05.

Finally, we confirmed the pro-survival effect of GSK-3 inhibition in the RP model with a third GSK-3 inhibitor, tideglusib. This compound has a chemical scaffold different to the other two inhibitors described above (compounds **1** and **2**) and shows a non-ATP, non-substrate competitive mechanism of GSK-3 inhibition. The treatment of *rd10* mouse retinal explants with tideglusib significantly reduced photoreceptor cell death (Figure 5), an observation that reinforces the role of GSK-3 as pharmacological target in retinal RP neuroprotection. Further, it opens an interesting translational opportunity. Tideglusib is an oral drug that has shown a wide safety window in human clinical trials both in Alzheimer’s disease and progressive supranuclear palsy^34^, and it is currently on clinical trials for autism spectrum disease^26^. In the light of the results described here, we propose the use of tideglusib for the treatment of retinal diseases, such as RP.

## Conclusions

During the last years a large body of evidence shows that GSK-3 pathway is altered in neurodegenerative conditions of the central nervous system and small molecules targeting GSK-3 selectively have been developed as disease-modifying drugs for several unmet diseases. However, the role of GSK-3 inhibitors in retinal pathologies has not been extensively studied although they may represent potential therapeutic candidates for retinal degeneration therapy. Hereby, we have shown that GSK-3 inhibition results in a protection of retinal ganglion cells and photoreceptor cells, an observation made with three different inhibitors in two different *ex vivo* models of retinal pathologies, including RP. The use of mechanistically and structurally diverse GSK-3 inhibitors allows us to state that the neuroprotective effect was produced by the target inhibition, highly reducing the possibilities of cross-reactivity based on the structure or inhibition type. The results of these studies suggest that GSK-3 inhibitors are a potential pharmacotherapeutic strategy to rescue photoreceptor cells, as well as retinal ganglion cells, from a progressive degeneration such the one present in RP. Furthermore, results obtained upon tideglusib treatment in the *rd10* model leads us to propose this compound as a clinical lead for RP.

Finally, the transient benefit observed in RP patients after oral intake of indirect GSK-3 inhibitor valproic acid, suggests and supports the clinical therapeutic potential of GSK-3 inhibitors for the treatment of RP. Together with the results here shown, selective GSK-3 modulation emerges as a new therapeutic target for the treatment of RP, opening new lines of research to discover an effective pharmacotherapy for RP and other retinal diseases.
